# High-Strain-Rate Deformation Behavior of Co_0.96_Cr_0.76_Fe_0.85_Ni_1.01_Hf_0.40_ Eutectic High-Entropy Alloy at Room and Cryogenic Temperatures

**DOI:** 10.3390/ma17122995

**Published:** 2024-06-18

**Authors:** Kun Jiang, Zhiping Xiong, Xi Chen

**Affiliations:** 1 Center for X-Mechanics, Zhejiang University, Hangzhou 310027, China; 2 ZJU-Hangzhou Global Scientific and Technological Innovation Center, Zhejiang University, Hangzhou 311215, China; 3 National Key Laboratory of Science and Technology on Materials under Shock and Impact, Beijing 100081, China

**Keywords:** eutectic high entropy, high-strain-rate deformation, deformation twinning, strain hardening

## Abstract

The deformation behaviors of Co_0.96_Cr_0.76_Fe_0.85_Ni_1.01_Hf_0.40_ eutectic high-entropy alloy (EHEA) under high strain rates have been investigated at both room temperature (RT, 298 K) and liquid nitrogen temperature (LNT, 77 K). The current Co_0.96_Cr_0.76_Fe_0.85_Ni_1.01_Hf_0.40_ EHEA exhibits a high yield strength of 740 MPa along with a high fracture strain of 35% under quasi-static loading. A remarkable positive strain rate effect can be observed, and its yield strength increased to 1060 MPa when the strain rate increased to 3000/s. Decreasing temperature will further enhance the yield strength significantly. The yield strength of this alloy at a strain rate of 3000/s increases to 1240 MPa under the LNT condition. Moreover, the current EHEA exhibits a notable increased strain-hardening ability with either an increasing strain rate or a decreasing temperature. Transmission electron microscopy (TEM) characterization uncovered that the dynamic plastic deformation of this EHEA at RT is dominated by dislocation slip. However, under severe conditions of high strain rate in conjunction with LNT, dislocation dissociation is promoted, resulting in a higher density of nanoscale deformation twins, stacking faults (SFs) as well as immobile Lomer–Cottrell (L-C) dislocation locks. These deformation twins, SFs and immobile dislocation locks function effectively as dislocation barriers, contributing notably to the elevated strain-hardening rate observed during dynamic deformation at LNT.

## 1. Introduction

High-entropy alloys (HEAs) along with medium-entropy alloys (MEAs) are collectively known as multi-principal-element alloys. Their unique alloy design concepts often give rise to distinctive atomic structure characteristics, manifesting a multitude of exceptional properties, especially in terms of mechanical properties [1,2,3]. These alloys typically display high strength, remarkable toughness and elevated strain-hardening rates, thereby garnering widespread attention and research focus globally [4]. Generally, HEAs and MEAs composed of a single face-centered cubic (FCC) phase, such as CoMnFeCoNi and CrCoNi alloy, display commendable ductility and strain-hardening ability but relatively low yield strength at different temperatures [5,6,7,8,9]. The yield strength of these alloys at room temperature is usually only about 200 MPa. Although lowering the temperature to LNT can greatly increase their yield strength, it will not exceed 500 MPa in most cases. In contrast, HEAs such as TaNbHfZrTi, characterized by a single body-centered cubic (BCC) phase, demonstrate higher strength but exhibit modest ductility [10]. Consequently, negotiating a balance between high strength and ductility in single-phase multi-principal-element alloys presents a significant challenge. Addressing this conundrum, Lu et al. [11] introduced the concept of eutectic HEAs (EHEAs). By combining the high strength of BCC HEAs and the high ductility of FCC HEAs, they successfully devised an FCC(B2)/BCC(L12) laminated-structure AlCoCrFeNi_2.1_ EHEA. This novel architecture embodies the idealized medley of superior strength and high ductility. Subsequently, a large number of new EHEA systems have been developed [12,13,14,15,16,17,18,19,20,21,22,23,24]. Integrating the advantages of eutectic alloys with those of single-phase HEAs, these EHEAs present manageable microstructures and desirable strength, complemented with commendable castability. This amalgamation of properties charters a fresh course for the mass-scale synthesis and broad-spectrum industrial application of high-entropy alloys, opening new avenues in the materials science domain.

EHEAs, esteemed as a prospective class of structural and functional materials, harbor considerable potential for deployment under severe conditions, notably in sectors such as aerospace, aeronautics, maritime and military. These domains typically demand materials resistant to the direst service conditions, encompassing high strain rates or complex environments of high strain rates coupled with cryogenic temperatures. Consequently, scrutinizing the mechanical properties and deformation mechanism of EHEAs in these harsh environments is a factor of decisive importance. Nonetheless, research on dynamic response mainly focuses on multi-principal-element alloys with a single FCC phase structure, such as CoCrFeNiMn, Al_0.1_CoCrFeNi, Al_0.3_CoCrFeNi and CrCoNi alloys [25,26,27,28,29,30,31,32,33,34]. Despite demonstrating considerable work hardening and impressive plasticity under dynamic load, the relatively low yield strength of these high-entropy alloys curtails their engineering effectiveness notably [33]. In contrast, despite its significant engineering potential, the exploration of the mechanical behaviors of EHEA under high strain rates is still in the early stages. Concurrently, studies predominantly focus on the mechanical properties in quasi-static conditions. Importantly, it must be acknowledged that strain rate could also considerably influence mechanical properties [35]. Metalliferous materials under high-strain-rate loading may exhibit localized shear instability behavior that diverges from static or quasi-static circumstances. Furthermore, when metalliferous materials withstand impact loads or are deformed at cryogenic temperatures, mechanical twinning, an auxiliary reinforcing mechanism, is more likely to be sparked, thus fostering an excellent balance between the material’s strength and ductility, also known as the twin-induced plasticity (TWIP) effect [36]. However, a study by Hu et al. on the dynamic deformation behavior of the AlCoCrFeNi_2.1_ EHEA did not detect any deformation twins [37].

In this work, we successfully prepared Co_0.96_Cr_0.76_Fe_0.85_Ni_1.01_Hf_0.40_ EHEA with a homogeneous and ultra-fine-layered microstructure. The dynamic compressive mechanical behaviors of this EHEA at room and cryogenic temperatures have been investigated using a split Hopkinson pressure bar (SHPB) furnished with a cryogenic system, and the microstructures of the alloy before and after deformation have been systematically characterized using a scanning electron microscope (SEM) and a transmission electron microscope (TEM). Interestingly, high-density stacking faults and nano-scale deformation twins have been observed in the FCC phase after high-strain-rate deformation. The strain rate sensitivity, deformation mechanism and strain-hardening characteristics of this EHEA under different loading conditions have been discussed in detail.

## 2. Experimental Methods

### 2.1. Material Preparation

First, (CoCrFeNi)_x_ was chosen as one part and (Co_a_Cr_b_Fe_c_Ni_d_Hf_e_) was chosen as another part for the current investigated alloy. When e is selected as 0.4, a, b, c and d will be randomly selected around the compositions (Co_0.11_Cr_0.18_Fe_0.37_Ni_0.55_Hf_0.4_) of the Laves phase in eutectic CoCrFeNiHf_0.4_. Following this rule, another part was chosen as Co_0.26_Cr_0.06_Fe_0.15_Ni_0.31_Hf_0.40_ in the current study. Second, we filtrated the compositions by varying x and examined whether the composition of (CoCrFeNi)_x_(Co_0.26_Cr_0.06_Fe_0.15_Ni_0.31_Hf_0.40_) was eutectic or not using the simple mixture method. Last, the eutectic compositions were identified to be Co_0.96_Cr_0.76_Fe_0.85_Ni_1.01_Hf_0.40_. Next, high-purity raw materials with purity greater than 99.99% were selected, and the Co_0.96_Cr_0.76_Fe_0.85_Ni_1.01_Hf_0.40_ EHEA used in this study was prepared using the vacuum arc melting method in an argon atmosphere. Each sample was remelted at least five times and magnetic stirring was added to ensure uniform composition. The melt was then sucked into a water-cooled cylindrical copper mold to acquire a button ingot with a diameter of ~40 mm and a thickness of ~15 mm. Finally, wire cutting was used to prepare cylindrical compression specimens with a size of Φ 5 × 5 mm for low- and high-strain-rate compression tests.

### 2.2. Mechanical Characterization upon Quasi-Static and Dynamic Tests

**Figure 1:**
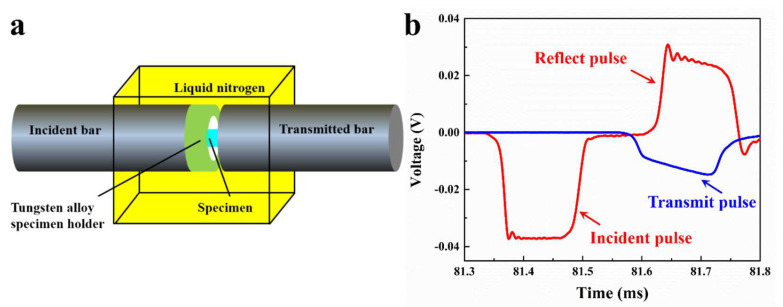
(**a**) Schematic illustration of the dynamic compression test at cryogenic temperature. (**b**) The typical waveform in a dynamic compression experiment.

Quasi-static compression tests with strain rates of 10^−3/^s and 10^−2/^s were conducted on a universal testing machine. Room-temperature (RT, 298 K) as well as liquid nitrogen-temperature (LNT, 77 K) high-strain-rate compression tests were conducted at strain rates of ~1000/s, ~2000/s and ~3000/s on the SHPB furnished with cryogenic equipment. As shown in Figure 1a, the specimen under investigation was positioned between the incident and transmitted bar to enable loading at high strain rates. The use of a tungsten alloy sample holder enabled the control of the specimen’s compression displacement through thickness adjustments, ensuring a single loading sample in the SHPB experiment was available for subsequent microstructural scrutiny. RT compression tests were executed under standard laboratory conditions. Conversely, during the tests at cryogenic temperatures, the sample was entirely submerged in liquid nitrogen, and retained for a duration of at least 5 min before being loaded. Figure 1b presents a representative waveform in a dynamic compression experiment. To corroborate the reliability of the experimental findings, all mechanical tests were performed a minimum of three times under each loading condition.

### 2.3. Microstructure Characterizations

The microstructure before and after deformation was characterized using a Zeiss Gemini 300 SEM (Zeiss, Oberkochen, Germany) attached to an electron backscatter diffraction (EBSD) system and a TEM equipped with an energy spectroscopy (EDS) system. The working voltage of the EBSD experiment was 20 kV. EBSD data were collected and processed using Aztec Crystal 2.1.2 software. In addition, nanoscale characterization was performed using FEI Talos F200X TEM equipment (Thermo Fisher Scientific, Waltham, MA, USA) at an operating voltage of 200 kV. The EBSD sample was first mechanically ground to 5000 grit with silicon carbide sandpaper, then polished to a mirror surface with silicon carbide polishing liquid, and finally the surface of the sample was argon-ion-polished using an argon ion polisher. The TEM samples were first polished with silicon carbide sandpaper to less than 50 microns, then cut into discs with a diameter of 3 mm, and finally precision-ion-thinned using a Gatan 697 Ilion instrument (Gatan, Pleasanton, CA, USA).

## 3. Results and Discussions

### 3.1. Initial Microstructural Characterization of the Prepared EHEA

**Figure 2:**
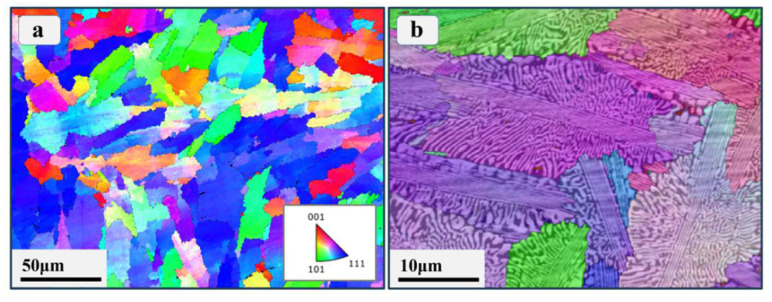
(**a**) The large-area EBSD inverse pole figure map of the prepared Co_0.96_Cr_0.76_Fe_0.85_Ni_1.01_Hf_0.40_ EHEA. (**b**) Higher magnification micrograph of the EBSD characterization for this EHEA.

**Figure 3:**
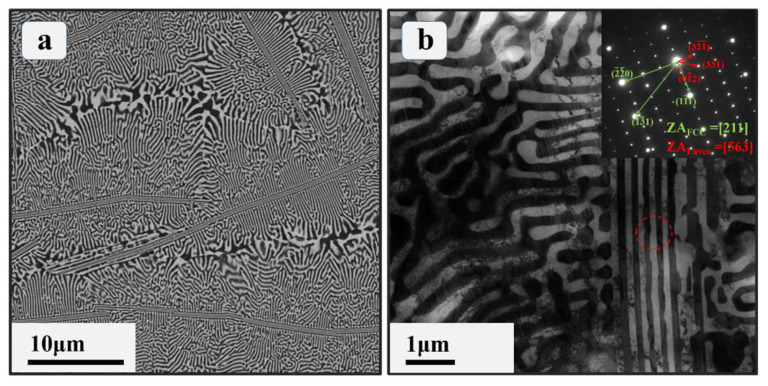
(**a**) The typical SEM micrograph of the prepared Co_0.96_Cr_0.76_Fe_0.85_Ni_1.01_Hf_0.40_ EHEA. (**b**) The typical TEM bright-field image of the prepared Co_0.96_Cr_0.76_Fe_0.85_Ni_1.01_Hf_0.40_ EHEA.

In order to fully understand the microstructure of the EHEA prepared at different scales, EBSD characterization was first carried out to exhibit the grain-scale information. Figure 2a displays the large-scale EBSD inverse pole figure map of the prepared Co_0.96_Cr_0.76_Fe_0.85_Ni_1.01_Hf_0.40_ EHEA. The mean grain size is approximately 20 μm for this EHEA. A closer examination of the higher magnification EBSD inverse polar figure map (as shown in Figure 2b) reveals a uniform eutectic lamellar microstructure within these grains, constituted by two distinct phases. A representative SEM image in Figure 3a provides a clearer depiction of these uniform distributed lamellar structures, which are mainly composed of irregular lamellar structures and regular strip lamellar structures. To further identify the two disparate crystal structures in the Co_0.96_Cr_0.76_Fe_0.85_Ni_1.01_Hf_0.40_ EHEA more distinctly, detailed TEM observations were carried out. Figure 3b shows a typical TEM bright-field image of the Co_0.96_Cr_0.76_Fe_0.85_Ni_1.01_Hf_0.40_ EHEA and the insets exhibit the selected area electron diffraction (SAED) patterns. It is obvious that the eutectic structure is composed of an FCC phase and a Laves phase, which is consistent with previous reports [13,14].

**Figure 4:**
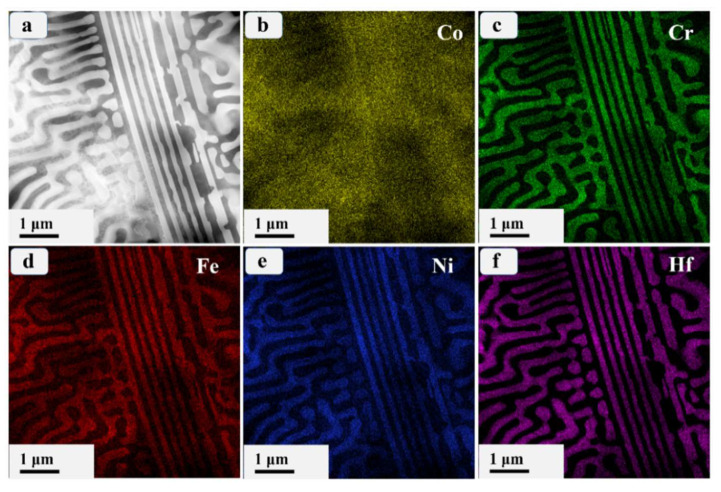
(**a**) The typical high-angle annular dark-field (HAADF) image and (**b**–**f**) corresponding elemental EDS maps of the prepared Co_0.96_Cr_0.76_Fe_0.85_Ni_1.01_Hf_0.40_ EHEA.

Figure 4a presents a representative high-angle annular dark-field (HAADF) image of the initial Co_0.96_Cr_0.76_Fe_0.85_Ni_1.01_Hf_0.40_ EHEA, in which the bright area is the Laves phase and the relatively dark area is the FCC phase. Figure 4b–f depict the TEM-EDS images of the corresponding region shown in Figure 4a. The observed elemental distributions, with Cr and Fe enrichment in the FCC phase and Ni and Hf enrichment in the Laves phase, agree with the existing literature [17,38]. The prevalent enrichment of two elements with a substantial mixing enthalpy in intermetallic compounds has also been documented [39,40]. This phenomenon is consistent with the current results, given that the mixing enthalpy of Hf and Ni in Laves is the largest, which is −42 kJ/mol [41]. Co distributes uniformly between the two phase, which has been reported in the literature [42].

### 3.2. Mechanical Properties

**Figure 5:**
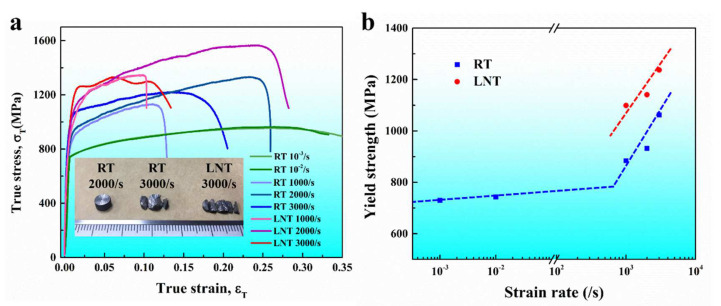
(**a**) The typical compressive true stress–strain curves of the Co_0.96_Cr_0.76_Fe_0.85_Ni_1.01_Hf_0.40_ EHEA under different strain rates and different temperatures. The insets exhibit the typical deformed specimens. (**b**) The variation in yield strength versus strain rate for the current EHEA—the prepared Co_0.96_Cr_0.76_Fe_0.85_Ni_1.01_Hf_0.40_ EHEA.

Figure 5a displays the quasi-static and dynamic compressive true stress–strain curves of the Co_0.96_Cr_0.76_Fe_0.85_Ni_1.01_Hf_0.40_ EHEA at different temperatures. The inset shows the pictures of the specimen after dynamic deformation under different typical conditions. Under quasi-static loading, the Co_0.96_Cr_0.76_Fe_0.85_Ni_1.01_Hf_0.40_ EHEA exhibits a high yield strength of ~740 MPa along with a high fracture strain of ~35%, indicating that the alloy has good plastic deformation ability under quasi-static conditions. In comparison to situations featuring low strain rates (quasi-static), the extant EHEA clearly exhibits a positive strain rate effect under conditions of elevated strain rates. At the strain rates of 1000/s, 2000/s and 3000/s, the yield strength of this alloy at RT increases to ~880 MPa, ~930 MPa and ~1060 MPa, respectively. Decreasing temperature will further enhance the yield strength significantly. Upon cooling the test temperature to LNT, the yield strength of this alloy at strain rates of 1000/s, 2000/s and 3000/s increases to ~1100 MPa, ~1140 MPa and ~1240 MPa, respectively. Under dynamic conditions, the loading specimen undergoes uniform deformation without damage when the strain rate does not exceed 2000/s. The stress drops on the stress–strain curves arose from stress wave unloading rather than damage to the specimen. For the dynamic deformation at the strain rate of 3000/s, macroscopic shear fracture occurred when the true plastic strain was less than 20% under RT conditions. Two cracks at approximately 45 degrees to the loading direction divided the cylindrical sample into three parts (the inset of Figure 5a). In addition, the fracture strain of the alloy decreased further to less than 15% when the temperature decreased to LNT, and the specimen broke into many smaller pieces (the inset of Figure 5a). Obviously, this alloy undergoes a ductile–brittle transition as the strain rate increases, and cryogenic temperature will expedite this transition.

Figure 5b elucidates the correlation between yield strength and strain rate, plotted on a logarithmic–logarithmic basis. Evidently, the dynamic strain rate sensitivity coefficient notably surpasses the quasi-static counterpart. The yield strength bifurcates pertaining to strain rate into two distinct zones: the thermally activated dislocation sliding area and the phonon drag-influenced dislocation movement area. Within quasi-static parameters, the dislocation velocity is significantly low, rendering the phonon drag effect negligible. An analysis of thermally activated dislocation motion shows that an increase in strain rate reduces the time for dislocations to move around obstacles, thereby diminishing the effect of thermal activation energy [31,43,44]. Consequently, dislocations necessitate a higher driving force to surmount obstacles. Within dynamic parameters, dislocation velocity is considerably higher than that of quasi-static conditions. High-velocity dislocations invariably induce phonon scattering, inciting an upsurge in dislocation kinematic viscosity. Moreover, fast-moving dislocations generate substantial amounts of heat, instigating local temperature alterations [30,31,43,45]. These incident sequences culminate in heat flow, which drains energy from the dislocations. Hence, dynamic dislocation motion is influenced by viscous drag, culminating in a strain rate sensitivity coefficient that considerably overshadows that in quasi-static conditions.

**Figure 6:**
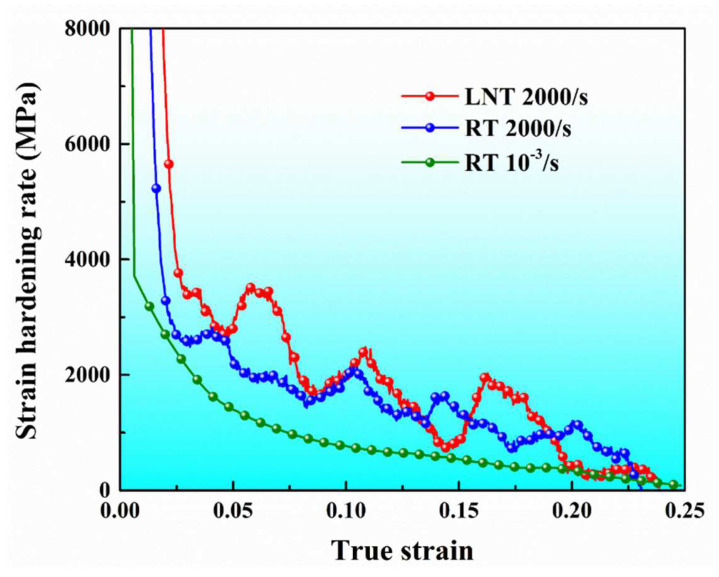
The representative strain-hardening-rate curves for the Co_0.96_Cr_0.76_Fe_0.85_Ni_1.01_Hf_0.40_ EHEA at different temperature.

Figure 6 illustrates the representative strain-hardening-rate curves for the Co_0.96_Cr_0.76_Fe_0.85_Ni_1.01_Hf_0.40_ EHEA at different strain rates and temperatures. Under the quasi-static condition, the strain-hardening rate of the alloy consistently declines with increasing plastic strain, indicating that the plastic deformation of the alloy is dominated by the dislocation slip mechanism. It is worth noting that the strain-hardening rate under dynamic conditions consistently surpasses that of quasi-static settings, and even a locally increased strain-hardening rate can be observed. This should mainly be attributed to the activation of new plastic deformation mechanisms besides dislocation slip, which will be analyzed in detail based on the characterization of the microstructure after deformation. Moreover, as the temperature decreases to LNT, the alloy’s strain-hardening rate increases further compared to RT, and a more pronounced localized increase in the strain-hardening rate can be discerned. This is fundamentally attributable to the genesis of high-density nanoscale deformation twins and Lomer–Cottrell (L-C) dislocation locks within the FCC region under LNT and high-strain-rate coupling conditions. This particular aspect will also be discussed in depth in the following section, based on the examination of the microstructure after dynamic deformation.

### 3.3. Microstructural Characterization after Dynamic Compression Tests

**Figure 7:**
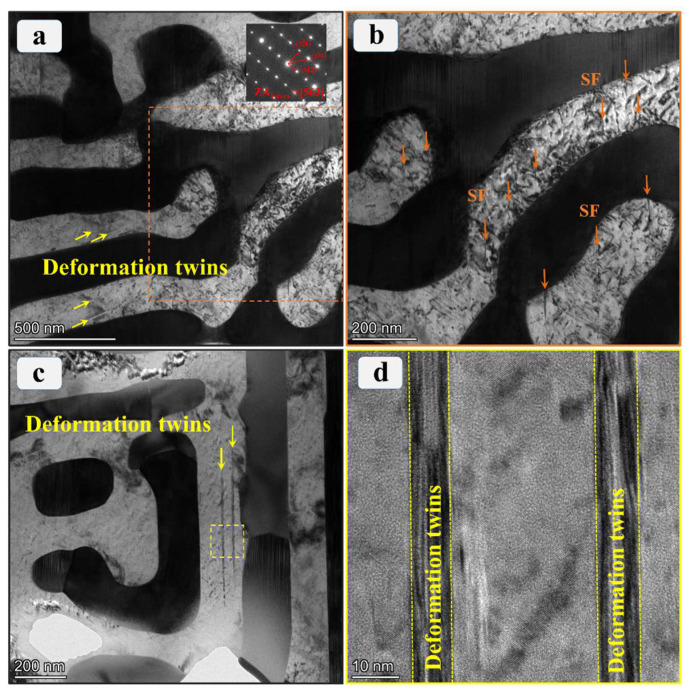
TEM microstructural characterizations of the Co_0.96_Cr_0.76_Fe_0.85_Ni_1.01_Hf_0.40_ EHEA after dynamic deformation at RT. (**a**) The TEM bright-field image showing high-density dislocation and several nanoscale deformation twins in the FCC phase region. The inset in (**a**) presents the SAED pattern of the Laves phase area. (**b**) Higher magnification image of dashed rectangular area in (**a**) exhibits high-density SFs. (**c**) The TEM bright-field image shows a few nanoscale deformation twins. (**d**) HRTEM image of the nanoscale deformation twins of yellow dashed rectangular area in (**c**).

The microscopic deformation mechanism of a material plays a pivotal role in shaping its macroscopic mechanical behavior. Therefore, a systematic TEM characterization of the dynamically deformed specimen was conducted to delve into the probable deformation mechanism of the Co_0.96_Cr_0.76_Fe_0.85_Ni_1.01_Hf_0.40_ EHEA under varied loading conditions. Figure 7 delineates the classic TEM micrographs of this alloy subsequent to dynamic compression deformation at RT. High-density dislocations appear conspicuous within the more compliant FCC phase, while, comparatively, the intrinsic dislocation density within the harder Laves phase is discernibly lower as illustrated in Figure 7a. Dislocation progression is restrained at the phase interface, instigating the accumulation of geometrically necessary dislocations (GNDs) along the phase boundary to sustain strain gradient and microstructural continuity amidst the FCC and Laves phases [45]. Figure 7b portrays the higher magnified TEM bright-field image of the region underscored by the orange dashed box in Figure 7a. It is apparent that in conjunction with high-density dislocations, the FCC phase additionally accommodates a sizable number of stacking faults (SFs). Numerous studies affirm that SFs can intersect with dislocations, functioning as a strong barricade against dislocation glide during deformation, hence augmenting strain hardening [30,46,47]. The manifestation of SFs is generally concurrent with the bifurcation of a full (a/2) <110> dislocation into twin (a/6) <112> Shockley partial dislocations [46]. High-strain-rate deformation may promote this process. It is worth noting that in the specimen after dynamic deformation at RT, a small number of nanoscale deformation twins can also be observed, as shown in Figure 7c,d. Extensive TEM exploration revealed that the density of deformation twins is very low. Therefore, during high-strain-rate compression deformation at RT, dislocation slip is the dominant deformation mode, but SFs and deformation twins will serve as new plastic deformation mechanisms to improve the strain-hardening ability of the alloy.

**Figure 8:**
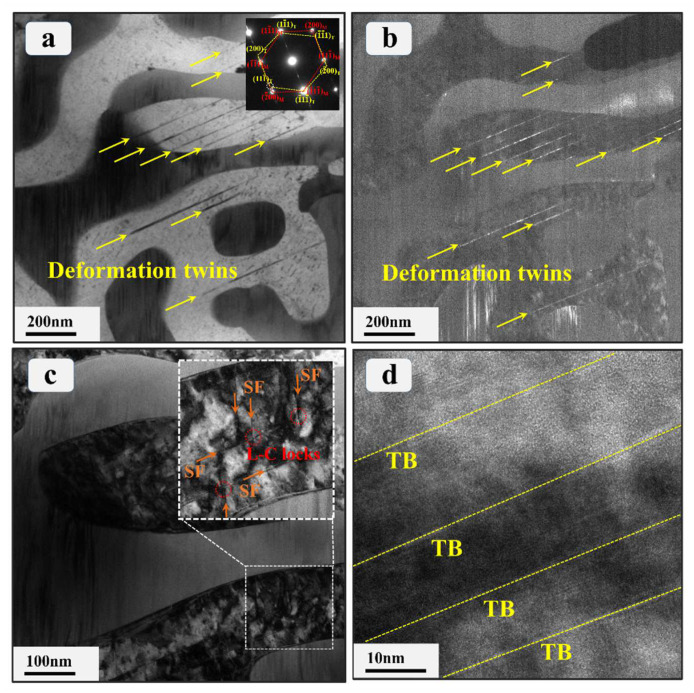
TEM microstructural characterizations of the Co_0.96_Cr_0.76_Fe_0.85_Ni_1.01_Hf_0.40_ EHEA after dynamic deformation at LNT: (**a**) The TEM bright-field image shows high-density nanoscale deformation twins. The inset in (**a**) presents the SAED pattern of the deformation twin region. (**b**) Corresponding TEM dark-field image highlighting the deformation twin lamellae. (**c**) An enlarged TEM bright-field image shows high-density SFs along two different slip directions. (**d**) HRTEM image of the nanoscale deformation twins.

Figure 8 illustrates the typical TEM micrographs of the Co_0.96_Cr_0.76_Fe_0.85_Ni_1.01_Hf_0.40_ EHEA corresponding to post-dynamic compression deformation under LNT conditions. A copious number of nanoscale deformation twins is discernible within the softer FCC phase (Figure 8a), which is significantly different from the specimen after dynamic compression at RT. Figure 8b reveals the TEM dark-field image associated with Figure 8a, in which the deformation twins are discernibly brighter, juxtaposed with the comparatively darker surrounding matrix. The width of the deformation twin lamellae is several to tens of nanometers. Figure 8c shows a higher magnification TEM bright-field image, which indicates a high density of dislocations prevalent within the FCC phase. Moreover, there are many SFs along two different directions. Figure 8d utilizes an HRTEM image to provide an atomic-scale focus on the deformation twins. The twin boundaries run parallel to the {111} plane of the FCC matrix. It is evident that in contrast to the dynamic deformation at RT, deformation twins and SFs also prevail as the primary plastic deformation mechanisms, along with dislocation slip. It is recognized that the twinning proclivity in FCC alloys is predominantly governed by stacking fault energy (SFE) [8,46,48,49,50]. Medium and low SFE facilities the formation of deformation twins [50]. Low SFE encourages the dissociation of a full dislocation into a Shockley partial dislocation, leading to the creation of SFs between these dislocations [46]. It has been documented that the SFE of alloys reduces with decreasing temperature [51]. Consequently, LNT conditions favor the dislocation dissociation and contribute to a higher density of deformation twins and SFs.

Interestingly, extensive L-C locks were discerned within the FCC region subsequent to dynamic deformation at LNT, as illustrated in Figure 8c, which exhibits a plethora of V-shaped SF interactions across two distinct directions. This V-shaped structure, constituted by two SFs of varied directions, is an archetypal attribute of L-C locks [46]. As per Yan et al. [52], L-C locks result from the interaction of Shockley partial dislocations on separate slip planes. The susceptibility to this interaction escalates owing to the heightened dislocation density in disassociation across two unalike slip planes, arising from the low SFE of the FCC phase in the Co_0.96_Cr_0.76_Fe_0.85_Ni_1.01_Hf_0.40_ EHEA. This, in turn, fosters an increased proclivity for the formation of these L-C locks. Several reports state that the reaction of two Shockley partial dislocations yields an immobile step dislocation that does not exist on the active {111} slip plane, consequently becoming stagnant [46]. Consequently, L-C locks substantially contribute to strain hardening by dislocation accumulation, a concept corroborated by former molecular dynamics (MD) simulations and experiments [53,54,55]. During LNT dynamic deformation, these resultant L-C locks lead to higher flow stress compared to RT deformation. Additionally, these dislocation locks, while acting as Frank–Read sources for dislocation multiplication, can hinder dynamic dislocation recovery induced by cross-slip, thus improving the efficacy and strain-hardening rate at LNT during dynamic deformation [46,56,57].

**Figure 9:**
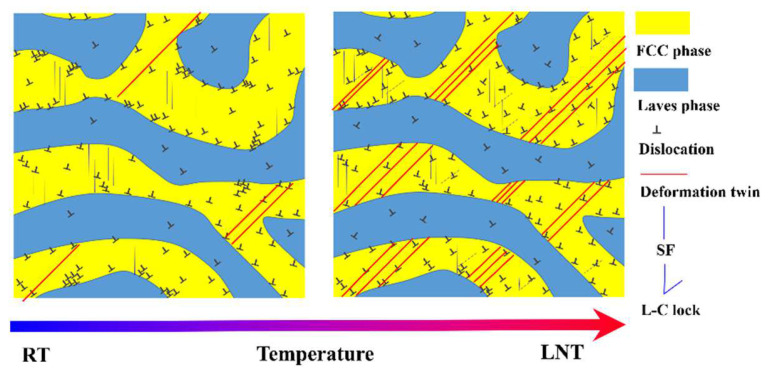
Schematic illustration exhibiting the dynamic deformation mechanisms of the Co_0.96_Cr_0.76_Fe_0.85_Ni_1.01_Hf_0.40_ EHEA under different temperature.

The schematic diagram of the dynamic deformation substructure of the Co_0.96_Cr_0.76_Fe_0.85_Ni_1.01_Hf_0.40_ EHEA is shown in Figure 9. At RT, although there are SFs and a small amount of deformation twins, the plastic deformation is dominated by dislocation slip. Furthermore, the FCC phase bears more plastic deformation. Under LNT conditions, the mechanism of dynamic deformation is influenced by a multistate interplay of dislocation slips, SFs and deformation twins. Twining and SFs as additional deformation modes are thought to contribute to increased strain-hardening rates. Additionally, a multitude of immovable L-C locks, originating from Shockley partial dislocation interactions, can function as dual-edged components, serving both as dislocation instigators and dislocation obstructions. This, in turn, incites an escalated strain-hardening rate in Co_0.96_Cr_0.76_Fe_0.85_Ni_1.01_Hf_0.40_ EHEA.

## 4. Conclusions

In this work, the compressive mechanical properties and underlying deformation mechanisms of the Co_0.96_Cr_0.76_Fe_0.85_Ni_1.01_Hf_0.40_ EHEA at varying strain rates and temperatures have been investigated thoroughly. SEM, EBSD and TEM characterization were carried out to carefully investigate the initial and deformed microstructures in this alloy. Several key conclusions can be summarized as follows:

(1) The current Co_0.96_Cr_0.76_Fe_0.85_Ni_1.01_Hf_0.40_ EHEA exhibits a high yield strength of 740 MPa along with a high fracture strain of 35% under quasi-static loading. A remarkable positive strain rate effect can be observed, and its yield strength increased to 1060 MPa when the strain rate increased to 3000/s. Decreasing temperature will further enhance the yield strength significantly. The yield strength of this alloy at strain rates of 3000/s increased to 1240 MPa under the LNT condition.
(2) The current EHEA exhibited a notable increased strain-hardening ability with either an increasing strain rate or a decreasing temperature. However, this alloy will undergo a ductile–brittle transition as the strain rate increases, and cryogenic temperature will expedite this transition.
(3) The analysis of microstructure after deformation suggests that the dynamic plastic deformation of the current Co_0.96_Cr_0.76_Fe_0.85_Ni_1.01_Hf_0.40_ EHEA at RT is dominated by dislocation slip. The deformation twins, SFs and immobile dislocation locks that emerge serve as effective dislocation impediments, thereby contributing to the pronounced strain-hardening rate during dynamic deformation at LNT.

## Data Availability

The original contributions presented in the study are included in the article, further inquiries can be directed to the corresponding author.
